# New Frontiers in Cancer Imaging and Therapy Based on Radiolabeled Fibroblast Activation Protein Inhibitors: A Rational Review and Current Progress

**DOI:** 10.3390/ph14101023

**Published:** 2021-10-05

**Authors:** Surachet Imlimthan, Euy Sung Moon, Hendrik Rathke, Ali Afshar-Oromieh, Frank Rösch, Axel Rominger, Eleni Gourni

**Affiliations:** 1 Department of Nuclear Medicine, the Inselspital, Bern University Hospital, University of Bern, CH-3010 Bern, Switzerland; surachet.imlimthan@extern.insel.ch (S.I.); hendrik.rathke@insel.ch (H.R.); ali.afshar@insel.ch (A.A.-O.); axel.rominger@insel.ch (A.R.); 2 Department of Chemistry—TRIGA Site, Johannes Gutenberg—University Mainz, 55128 Mainz, Germany; emoon@students.uni-mainz.de (E.S.M.); frank.roesch@uni-mainz.de (F.R.)

**Keywords:** fibroblast activation protein, cancer-associated fibroblast, tumor microenvironment, fibroblast activation protein inhibitor, nuclear imaging, radiotherapy

## Abstract

Over the past decade, the tumor microenvironment (TME) has become a new paradigm of cancer diagnosis and therapy due to its unique biological features, mainly the interconnection between cancer and stromal cells. Within the TME, cancer-associated fibroblasts (CAFs) demonstrate as one of the most critical stromal cells that regulate tumor cell growth, progression, immunosuppression, and metastasis. CAFs are identified by various biomarkers that are expressed on their surfaces, such as fibroblast activation protein (FAP), which could be utilized as a useful target for diagnostic imaging and treatment. One of the advantages of targeting FAP-expressing CAFs is the absence of FAP expression in quiescent fibroblasts, leading to a controlled targetability of diagnostic and therapeutic compounds to the malignant tumor stromal area using radiolabeled FAP-based ligands. FAP-based radiopharmaceuticals have been investigated strenuously for the visualization of malignancies and delivery of theranostic radiopharmaceuticals to the TME. This review provides an overview of the state of the art in TME compositions, particularly CAFs and FAP, and their roles in cancer biology. Moreover, relevant reports on radiolabeled FAP inhibitors until the year 2021 are highlighted—as well as the current limitations, challenges, and requirements for those radiolabeled FAP inhibitors in clinical translation.

## 1. Introduction

Cancer is a heterogeneous disease formed within an extremely complex microenvironment [1]. In fact, a malignant tumor does not only consist of cancerous cells but also a vast majority of endogenous host stromal cells (e.g., fibroblasts, and vascular and immune cells) and extracellular matrix (ECM) components, collectively known as the tumor microenvironment (TME) [2]. The TME is a unique milieu constituted within the tumor and can influence tumor development, immune evasion, metastasis, and therapeutic resistance through complex heterotypic interactions with the cancer cells [3]. The stromal cells in the tumor (tumor stroma) are the largest portion of the total tumor mass (over 90%) connected through desmoplastic reaction [4]. Recently, TME components and ECM remodeling have attracted great attention as crucial oncological factors that determine the behavior of cancer cells and disease progression [5]. Among all cells within the TME matrix, fibroblasts are considered to be dominant cells that have a strong association of their biological functions to all stages of cancer progression and metastasis [6]. Cancer-associated fibroblasts (CAFs), a type of continuously activated fibroblast, have been implicated to have a strong tumor-modulating effect and are commonly found in most solid tumors—such as breast, prostate, and pancreatic cancers [7,8]. Generally, CAFs account for up to 80% of all fibroblasts in the TME [9]. The protein molecules secreted by CAFs—such as growth factors, chemokines, cytokines, and matrix metalloproteinases—play a pivotal role in tumorigenesis through the stimulation of cell–cell communication and ECM remodeling [10]. In this light, CAFs have been demonstrated to be a potential biological target for cancer diagnosis and therapy based on the TME targeting approach [11]. CAFs are identified by the expression of various specific biomarkers on their surface, thus providing the opportunity to be used as a particular site for targeted radio- diagnostic and/or therapeutic applications. Among those, surface bound biomarkers characterized by neutral pro-tumorigenic or tumor-suppressive ability are α-smooth muscle actin (α-SMA), fibroblast-specific protein 1 (S100A4 or FSP-1), platelet-derived growth factor receptors (PDGFRα/β), and fibroblast activation protein (FAP) [12]. In the field of nuclear medicine in particular, FAP appears to be a promising target due to its non-expression in normal fibroblasts and the stroma of benign epithelial tumors compared to its significantly high accumulation, mainly on the stromal compartments of a variety of malignant tumors [13].

Nuclear imaging modalities, including positron emission tomography (PET) and single-photon emission computed tomography (SPECT), are noninvasive imaging tools widely used in the clinic. Tumor imaging using PET and SPECT techniques allows real-time monitoring and determination of target occupancy, pharmacokinetics, biodistribution, elimination, and treatment responses of established radiopharmaceuticals in vivo [14]. Furthermore, when the imaging agent that identifies the malignant lesions is followed by the administration of the companion therapeutic agent that treats the same lesions, the generated pair is integrated into the theranostic platform. The visualization of FAP-expressing fibroblasts has been investigated with different diagnostic as well as therapeutic radiolabeled agents, which could be grouped into three main categories: antibodies, FAP inhibitors (FAPIs) of low molecular weight, and peptidomimetic structures [15,16,17,18,19,20]. Although imaging of FAP showed great promise in both preclinical studies and clinical trials, the therapeutic efficacy of the fibroblast activation protein inhibitor alone is still limited due to a relatively short retention time of the corresponding radiopharmaceuticals in the tumor area. Therefore, the current effort has been directed towards the optimization of the molecular structure of FAPI to exhibit favorable pharmacokinetic performance, resulting in prolonged retention at the tumor site. Ultimately, this could provide a new convenient avenue for the development of FAP-based radiopharmaceuticals for the diagnosis and treatment of cancers predominantly.

In this review, we aim to provide a comprehensive overview of the biological functions and fates of TME, CAFs, and FAP in cancer prognosis and development. Besides, we further discuss the recent reports of FAP-based radiopharmaceuticals for nuclear imaging and targeted radionuclide therapy (endoradiotherapy) in the preclinical level and also provide a report on the current status with respect to the clinical assessment.

### Tumor Microenvironment: A New Arena in Stromal Targeting

**Figure 1:**
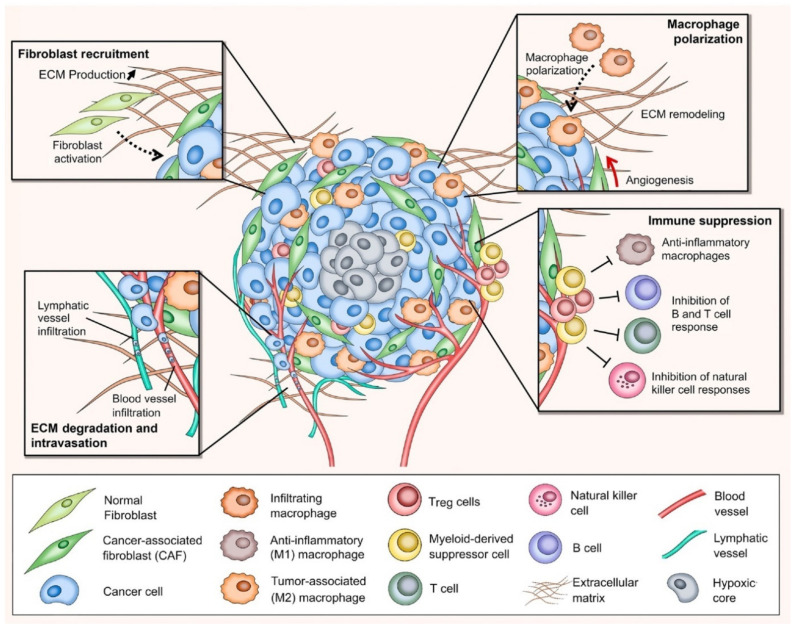
Schematic representation of the tumor microenvironment (TME) depicting various non-malignant and malignant cells, ECM compositions, and crucial biological processes developed within the TME. CAF: cancer-associated fibroblast; ECM: extracellular matrix; Treg: regulatory T cell; M1: anti-inflammatory macrophage; M2: tumor-associated macrophage. The figure is reproduced with permission from [23].

In the past decade, tremendous progress in understanding the role of TME participating in cancer development and growth has been achieved, facilitating the broadening of tumor targeting and cancer therapeutic approaches [21]. As previously mentioned, the TME comprises different types of stroma (non-malignant) cells (e.g., various phenotypes of fibroblasts, immune and inflammatory cells), ECM components, lymph nodes, nerves, and blood vessels (Figure 1). Within the TME, the interaction between tumor stroma (non-neoplastic part of the TME) and cancer cells is known to be the key parameter to direct tumor development and metastasis [22]. The activated stroma undertakes critical roles in cell invasion, extravasation, migration, angiogenesis, immunosurveillance evasion, and therapeutic resistance [23].

**Figure 2:**
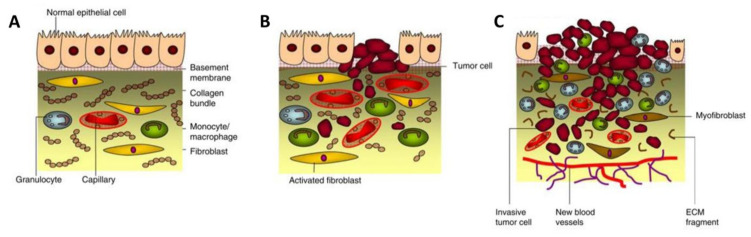
Tumorigenesis. (**A**) Normal epithelium with stromal compartment, including normal epithelial cells, basement membrane, macrophages, fibroblasts, and general ECM components. (**B**) Dysplasia; a process during tumorigenesis where the modified epithelial and stromal cells start excessively propagating and mutating due to cancer invasion. At this stage, the fibroblasts become activated, and macrophages are decreased. (**C**) Carcinoma; the ECM is degraded while the angiogenesis is formed to supply nutrients and oxygen to the tumor area, leading to the continual unregulated proliferation of cancer cells. The figure is reproduced and adapted with permission from [24].

The biological functions of the TME in cancer initiation and progression have been considered to be prominent for enhanced molecular-based diagnostic and therapeutic agents [24]. The tumor-associated stromal components in the TME are known to be the primary support of nutrient supplies for the establishment of metabolic networks with tumor cell compartments. Typically, the stromal cells secrete growth factors, chemokines, cytokines, miRNA, and extracellular vesicles in order to interact with cancer cells, the ECM components, and among themselves, leading to cancer metabolism involved in tumor development and progression [25]. The upregulation in the expression of oncogenes and proteins promotes additional oncogenic signaling pathways necessary for cell communication within tumor stroma, enhancing the proliferation and invasion of cancer cells [5,26,27]. In general, tumor stroma and cancer cells should have at least four critical capabilities to advance tumor development and progression, including mobility, ECM degradability, survival in blood circulation, and the ability to adapt and develop in a new tissue environment [24]. To acquire those traits, cancer cells employ the transcriptional factors (TFs), regulatory factors in orchestrating gene expression during the course of cancer development [28]. Recent studies demonstrated the potential of TFs to induce TME remodeling as well as governing the proliferation and migration of cancer cells [28,29,30]. To this end, the TME transmits the oncogenic signals to activate the TFs. The stromal cells induce the transcription programs allowing mesenchymal stem cells (MSCs) to invade distant tissues and create a new TME, after which the cancer cells shut down those transcriptional processes and reconvert the MSCs into epithelial cells while replicating themselves in the core of the tumor [24,31]. Therefore, tumorigenesis occurs from the abnormal development of cells within tissues before turning into a malignancy (Figure 2). Cancer cells secrete diverse growth factors and degrading proteinases, as well as stimulating the host to release biomolecules that can degrade the ECM and its component adhesion molecules. The degradation usually occurs at the surface of the tumor cell where there is an outbalance between degradative enzymes and natural proteinase inhibitors [24,32,33]. These secreted proteins and enzymes by tumor cells are involved in cell adhesion, cell signaling, cell motility, and invasion. When the ECM (i.e., collagen) is degraded, activated fibroblasts, inflammatory cells, and angiogenesis are triggered, thus resulting in the generation of growth factors and degrading enzymes, which are beneficial for cancer development and progression [24,34]. Taken together, the TME is considered as a target-rich milieu for cancer diagnosis and therapy as asserted by extensive investigations conducted over the past decade.

## 2. Cancer-Associated Fibroblasts: A Critical Mediator in Cancer Progression

**Figure 3:**
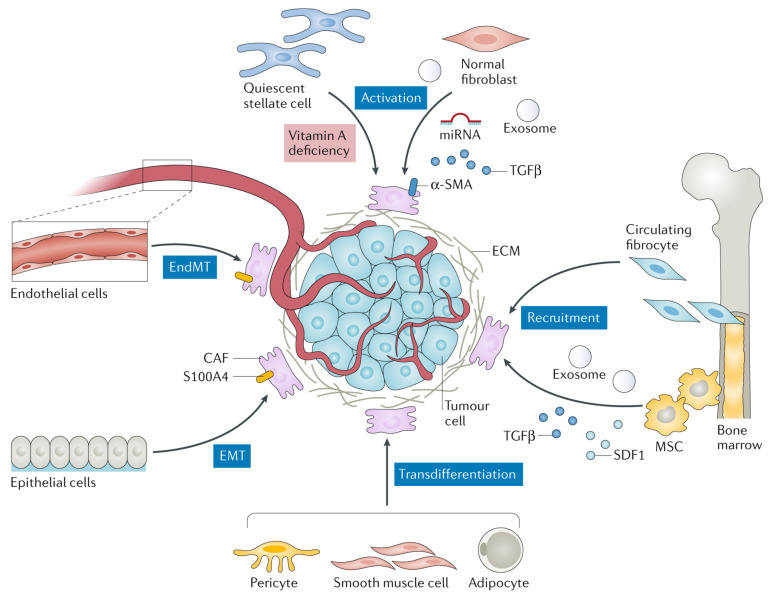
Potential cellular sources and biological processes involved in CAF formation. CAFs can be generated from normal resident fibroblasts (by activation), endothelial cells (by EndMT), epithelial cells (by EMT), pericyte/smooth muscle cell/adipocyte (by transdifferentiation), and circulating fibrocytes (by recruitment). EndMT is known as endothelial-to-mesenchymal transition, EMT as epithelial-to-mesenchymal transition, SDF1 as stromal-derived factor 1, S100A4 as fibroblast-specific protein 1, MSC as mesenchymal stem cell, TGFβ as transforming growth factor-β, and α-SMA as α-smooth muscle actin. The figure is reproduced with permission from [41].

Among all the stromal cells, cancer-associated fibroblasts (CAFs) are dominant populations in the TME structure. In general, fibroblasts are quiescent and become activated during wound repair and regeneration, collectively known as myofibroblasts [35]. In the normal wound healing cycle, myofibroblasts appear in the early phase of granulation tissue formation, then become the most abundant cell type in the proliferation phase before progressively disappear in the later stage of the wound healing by apoptotic mechanism [36]. Malignant tumors are conversely recognized as “wounds that do not heal” [37,38], prompting the activated fibroblasts (CAFs in this case) to become an excellent target for cancer treatment [39]. Several recent studies have revealed the critical participation of CAFs in cancer progression as well as modulation of an antitumor immune response [34,40,41,42]. In tumor stroma, CAFs appear as spindle-shaped cells widely distributed in most connective tissues and responsible for establishing and remodeling the ECM architecture. Although the exact origin, subtypes, and biology of CAFs are not well defined due to their heterogeneity and enigmatic cellular components [38,43], increasing investigation of CAFs in cancer indicates their significance in tumorigenesis, especially in solid tumors [34]. CAFs establish a host response to neoplastic transformation and present dynamic pro- and anti-tumorigenic features during cancer progression. They also exhibit similar functions as fibrosis-associated fibroblasts, such as epigenetic programing [44]. Due to their heterogeneity, CAFs can be recruited and activated through different biological pathways from various cellular sources (Figure 3), for instance, normal fibroblasts, bone marrow-derived fibrocytes (BMDFs), mesenchymal stem cells (MSCs), endothelial cells, epithelial cells, pericyte, smooth muscle cells, and adipocytes [45]. Within the local source (i.e., TME), cancer cells can induce the activation of those recruited normal resident fibroblasts into CAFs through the release of miRNA, exosome, and transforming growth factor-β (TGFβ). Various studies have demonstrated that quiescent pancreatic and hepatic stellate cells appear to have a myofibroblast-like phenotype upon activation, which means they are considered as CAFs in pancreatic and liver cancers [46,47,48].

Furthermore, BMDFs and MSCs may participate in the CAF pool triggered within the tumor stroma through the recruitment process. During the wound healing, BMDFs can migrate to the site of the inflammatory tissue (tumor area in this case) and differentiate into CAFs, contributing to tumor proliferation [49,50]. On the other hand, MSCs—another suggested CAF precursor—can undergo differentiation due to the excessive production of α-SMA responding to the TGFβ secreted by cancer cells for immunosurveillance evasion [51,52]. CAFs can also be differentiated through endothelial and epithelial cells through endothelial-to-mesenchymal transition (EndMT) and epithelial-to-mesenchymal transition (EMT), respectively. EndMT and EMT can directly polarize endothelial and epithelial cells to differentiate into mesenchymal before transforming into CAFs stimulating by TME secreted factors (e.g., S100A4, growth factors, and cytokines) [53,54,55]. The least common procedure in CAF differentiation is transdifferentiation (lineage reprogramming) where one mature somatic cell is converted into another somatic cell without complete dedifferentiation [56]. The cells that undergo transdifferentiation include adipocytes, smooth muscle cells, and pericytes [41].

Because of phenotypic heterogeneity in CAFs, the biological markers are diverse but exhibit specific expression patterns following local TME conditions [34]. CAFs are commonly identified based on the expression of various biomarkers, such as α-SMA, S100A4, platelet-derived growth factor receptors (PDGFRα/β), fibroblast activation protein (FAP), tenascin C, vimentin, and podoplanin (PDPN). While the expression of these markers is found on CAFs, several of them also reveal their expression in other cell types (e.g., immune cells, epithelial cells, non-mesenchymal ECM producing cells, lymphatic endothelial cells, and adipocytes), which also determines the functional heterogeneity of CAF biomarkers [34,41,42]. In fact, CAF biomarkers have been intensely explored but still lack precise identification of specific expression patterns with well-defined features [57]. In particular, α-SMA, FAP, and PDGFRα/β are highly expressed in CAFs and have been extensively used in several oncological investigations to determine CAF populations. Among them, FAP has received tremendous interest as a potential biomarker for CAF identification and targeting due to its overexpression in most of the cancer types but low to undetectable in normal fibroblasts presented in the body [11,58,59,60]. The most relevant CAF biomarkers and their biological functions are compiled in Table 1.

## 3. Fibroblast Activation Protein: A Potential Diagnostic and Therapeutic Target across Stromal Barriers

### 3.1. Biological Characteristics of FAP

**Figure 4:**
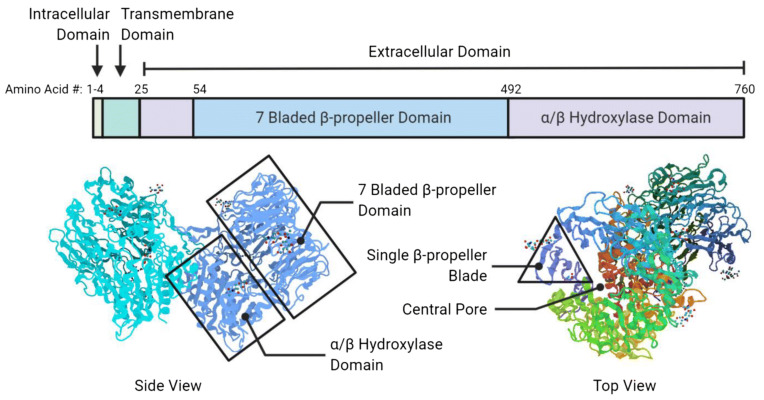
Schematic representations of FAP structural compositions (top panel) and its protein scaffold (bottom panel). In the protein scaffold, the central pore, seven-bladed β-propeller, single β-propeller, and α/β hydroxylase domains are identified. “#” represents the position of the amino acid residue in FAP domains. The figure is reproduced with permission from [13].

Fibroblast activation protein alpha (FAPα or FAP), also known as prolyl endopeptidase FAP or seprase, is a type II membrane-bound serine protease (97 kDa subunit) associated with fibrosis, tissue repair, inflammation, and ECM degradation [57]. Importantly, FAP is highly expressed in CAFs, a major constituent of tumor stroma, and is upregulated in more than 90% of human epithelial cancers [61,62]. Due to this reason, FAP is widely recognized as a crucial biomarker to identify potential CAF-positive tumor stroma. FAP contains 760 amino acids in its structure in which residues 1–4 are in the intracellular domain, residues 5–25 in the transmembrane domain, and residues 26–760 in the extracellular domain (Figure 4). Within the extracellular domain, the β-propeller domain comprises the amino acid residues 54–492 (i.e., substrate selectivity gate) while the residues 26–53 and 493–760 belong to the α/β hydroxylase domain [13,63].

FAP is the homolog of dipeptidyl peptidase IV (DPPIV or CD26), one of the members of the prolyl peptidase family. DPPIV shares about 50% similarity in amino acid sequence with FAP, and 70% homology of the catalytic domain [64,65,66]. FAP contains two types of enzymatic activity: dipeptidyl peptidase and endopeptidase. Unlike FAP, DPPIV does not display endopeptidase activity. The endopeptidase allows FAP to mediate the proteolytic processing of matrix metalloproteinase-cleaved collagen I, leading to the prevention of morphogenesis, tissue remodeling, and repair [67,68,69]. Therefore, FAP-specific detection has been directed towards the endopeptidase activity as well as the development of novel FAP-targeted inhibitory molecules [13,70]. Through enzymatic and non-enzymatic activities, FAP demonstrates pro-tumorigenic activity involved in migration, invasion, and proliferation of stromal fibroblasts, immune, endothelial, and cancer cells, resulting in ECM degradation, tumor angiogenesis, invasiveness, and immunosurveillance evasion [71,72,73].

In general, the FAP monomer is considered inactive but can exhibit activity in the form of homodimers and heterodimers with DPPIV [74]. For the regulation of FAP activity, dimerization and glycosylation are required. The homodimer (activated FAP) can assemble into a heterodimer by merging FAP and DPPIV, which participates in the fibroblast migration to the collagenous matrix [75]. FAP can also bind to β-integrins, the important proteins for cell adhesion, signal transmission, and activation of cellular response. This process provides an enhanced cellular localization of FAP in the actin-rich protrusions on the plasma membrane of malignant cells, which involves the ECM degradation of cancer invasiveness [76]. Besides, glycosylation is essential for the endopeptidase activity of FAP for which five potential *N*-linked glycosylation sites are identified on asparagine residues in both β-propeller (49, 92, 227, 314) and α/β hydroxylase (679) domains [13,77].

### 3.2. Relationship between FAP and Immunosuppression in the TME

The complex interactions between stromal and cancer cells in the TME vastly contribute to carcinogenesis and tumor progression. However, the high immune tolerance of tumors raises complexity and impediment in cancer immunotherapy [65]. The immune cells within the TME are immunosuppressive cells, such as tumor-associated macrophages (TAMs), myeloid-derived suppressor cells (MDSCs), natural killer (NK) cells, cytotoxic CD8 T cells, and regulatory T (Treg) cells [78]. Unlike normal tissues, immune cells in the TME are significantly low in number and are inactive, which paves the way for cancer’s camouflage from the immunosurveillance and the attack of effector cells, leading to ineffective treatment. Growing evidence has suggested that FAP is one of the immunosuppressive components in the TME that induces tumor-promoting inflammation [79]. Feig et al. reported the mediation of immune suppression by chemokine CXCL12 from FAP-expressing CAFs in pancreatic cancer. The studies showed that the administration of AMD3100 (CXCL12 inhibitor) induced rapid T-cell accumulation in the region of tumor-containing cancer cells and performed synergistically with α-programmed cell death 1 ligand 1 (α-PD-L1) to greatly induce the apoptosis of cancer cells. Hence, the CXCL12 protein secreted by FAP-positive cells may direct the immunosuppression in human pancreatic ductal adenocarcinoma [80]. Furthermore, a similar approach was investigated using an oncolytic virus-induced T-cell accumulation. The oncolytic group B, adenovirus enadenotucirev, was first modified to express a stroma-targeted bispecific T-cell engager (BiTE) to specifically bind to FAP on CAFs and CD3 epsilon protein on T cells in malignant ascites and solid prostate cancer biopsies. With the FAP-BiTE encoding virus, tumor-infiltrating PD-L1 positive T cells were induced to obstruct CAFs. In ascites, this resulted in depletion of immunosuppressive factors and increased T-cell function and trafficking [81]. Recently, FAP protein expression in 92 colorectal cancers was determined using transcriptomic and immunohistochemical data. The observation showed that FAP expressing genes were upregulated in both mRNA and protein levels and had a high association with immune cells. Moreover, the abundance of Treg cells within the tumor region was observed, as well as the depletion of helper T (Th1) cells and NK cells, indicating an immunosuppressive environment induced by secreted components from FAP positive cells [82]. However, the FAP mechanism on immunosuppression is still in its infancy and needs further investigation to determine the exact role in the suppression of the antitumor activity of immune cells in the TME [65,74].

### 3.3. FAP as a Potential Target in Cancer 

As previously described, FAP expression is extremely low to absent in normal tissues while it is overexpressed in more than 90% of human cancers, such as breast, colorectal, pancreatic, melanoma, myeloma, gastric, brain, and ovarian carcinomas [62,82,83,84,85,86,87]. Thus, FAP is highly expressed in CAFs within tumor stroma, along with its fast and efficient internalization, rendering as an attractive biomarker. Although the biological mechanism of FAP on cancer prognosis is still vague and inconsistent throughout reports in the literature, the existence of FAP in malignant stroma is determinative as a promising target for cancer imaging and therapy [11,14]. FAP targeting leads to degradation of the ECM, interfering in regulatory signaling and subsequently disrupts the supportive biological functions of stromal CAFs on the tumor growth [63,88]. As a part of the ongoing efforts to develop FAP targeting agents, several ligands have been reported. They mainly fall into three categories: antibodies, FAP inhibitors, and peptides. 

First, human FAP was originally identified in cultured fibroblasts using the monoclonal antibody (mAb) F19 [89]. Sibrotuzumab/BIBH1, a humanized version of the F19 antibody as well as other humanized or fully human antibodies against FAP antigen exhibiting specificity towards the F19 epitope have been reported [90,91]. OS4 is another humanized antibody (CDR-grafted) derived from the F19 antibody [92]. Furthermore, murine anti-FAP antibodies, including chimeric and humanized versions have been evolved [93]. 

A second strategy aiming at FAP targeting is based on the inhibition of enzymatic activity using small molecule inhibitors. Findings, mainly derived from preclinical studies, suggest that the inhibition of FAP-enzymatic activity induced by low molecular weight inhibitors has the potential to decrease the invasiveness of malignant cells and further lead to a considerable reduction of the tumor growth [94]. The design of FAP-specific inhibitors remains challenging due to the homology of enzymatic substrate domains shared with other dipeptidyl peptidase members; therefore, a precise characterization of FAP substrate and inhibitor matching is required for the structural design of novel synthetic FAPIs to specifically target the endopeptidase activity domain in FAP [74,95]. Val-boroPro (talabostat, PT-100) is a non-selective boronic acid-based inhibitor that targets on both FAP and DPPIV enzymatic domains. Talabostat has shown promising preclinical results but demonstrated suboptimal results in the phase II clinical trial in patients with metastatic colorectal cancer [96]. Moreover, the combination of talabostat with chemotherapeutic drugs, such as docetaxel and cisplatin, was conducted in non-small cell lung cancer and metastatic melanoma patients, respectively. However, the results were unsuccessful to demonstrate significant therapeutic outcomes in phase II clinical trials due to safety and efficacy reasons [97,98]. On the other hand, d-Ala-boroPro-based FAP inhibitor exhibits selectivity towards other dipeptidyl peptidase members compared to FAP by a factor of 40 [99]. However, no in vivo pharmacokinetic data for this inhibitor has been reported [100]. Linagliptin, a dual FAP and DPPIV inhibitor, has also shown a worthwhile effect in inhibiting the FAP enzymatic activity [101]. Furthermore, pyroglutamyl(2-cyanopyrrolidine) and quinolinoylglycyl(2-cyanopyrrolidine) derivatives have demonstrated highly satisfactory selectivity to FAP over other dipeptidyl peptidase members [99,102]. 

Thirdly, a FAP binding peptide coupled to the radionuclide chelator DOTA (1,4,7,10-tetraazacyclododecane-1,4,7,10-tetraacetic acid)—which could serve as a potential radiotherapeutic tracer named FAP-2286—was evaluated in both preclinical and clinical levels. FAP-2286 revealed promising data with respect to its potency, selectivity, and efficiency towards FAP [19,20].

## 4. Development of Radiolabeled-Based FAP Tracers for Tumor Stroma Mediated Nuclear Imaging and Radionuclide-Based Therapy 

FAP targeting using FAP-based radiolabeled tracers allows the delivery of radionuclides carrying either imaging photons and/or ionizing particles (α and β^+^/β^−^) directly to tumor stroma, resulting in nuclear imaging and/or radionuclide therapy of FAP-positive tumors. Compared to the flagship PET radiotracer, [^18^F]FDG, widely used in the clinic, FAP-based targeting radiolabeled inhibitors may provide an alternative strategy in nuclear molecular imaging for detecting tumors with low or heterogeneous glucose metabolism as well as those located close to highly glycolytic tissues to avoid non-specific uptake that could cause a high background signal [88]. As stroma occupies a major part in the tumor volume, FAP-targeted radiotracers may increase the target sensitivity and image contrast of the disease area compared to the targeting of glucose metabolic pathway in cancer cells solely due to their lack of brain uptake in contrast to [^18^F]FDG. Nevertheless, the available reports on preclinical and clinical assessments of radiolabeled FAPIs are still in an early phase, warranting further research and development. Recently, different radiolabeled FAP-based tracers have been examined for noninvasive nuclear imaging and targeted radionuclide therapy. In the following section, relevant FAP-targeted radioligands developed over the past few years are highlighted. 

### 4.1. Radiolabeled FAP-Targeted Antibodies

Because of the consistent presence of FAP on the tumor stroma and the accessibility of FAP-positive tumor stromal fibroblasts to circulating monoclonal antibodies (mAbs), several studies have suggested possible diagnostic and therapeutic applications of humanized mAb and their constructs with novel immune and nonimmune effector functions. The murine F19 mAb, which recognizes FAP labeled with ^131^I, has been used for that purpose [89]. Two phase-I quantitative biodistribution studies with [^131^I]I-F19 mAb in patients with hepatic metastasis from primary colorectal cancer and soft tissue sarcoma have demonstrated the proof of principle of stromal targeting [89,103]. Based on the results acquired with the use of the murine F19 mAb and aiming at addressing the problems that appeared due to the immune responses to murine antibodies, a humanized version of F19—named sibrotuzumab—was preclinically and clinically investigated after radiolabeling with ^131^I. [^131^I]I-sibrotuzumab was found to preferentially bind to FAP in vitro in the same manner as its murine counterpart without affecting the FAP-related enzymatic activity. Besides, the results of the first-in-human clinical study demonstrated the ability of [^131^I]I-sibrotuzumab to target stromal FAP and provided evidence that sibrotuzumab has a considerable promise in the targeting and therapy of epithelial malignancies. However, [^131^I]I-sibrotuzumab failed to provide a measurable therapeutic activity in phase I/II clinical trials despite excellent tumor stroma targeting properties. In addition, about one-third of the sibrotuzumab treated patients developed human-anti-human antibodies (HAHA) and a reduction in tumor uptake. Therefore, further clinical development of sibrotuzumab has been discontinued [104]. In an attempt to address the side effects caused by sibrotuzumab, the successful selection of two human mAbs (ESC11 and ESC14) from a phage-display library took place. Both ESC11 and ESC14 presented the advantage of being rapidly internalized by FAP-positive cells. The labeling with the β-emitting ^177^Lu of both exhibited specific accumulation in FAP-positive human melanoma xenografts, providing a delay in tumor growth in vivo [105].

### 4.2. Radiolabeled FAP-Based Inhibitors

**Figure 5:**
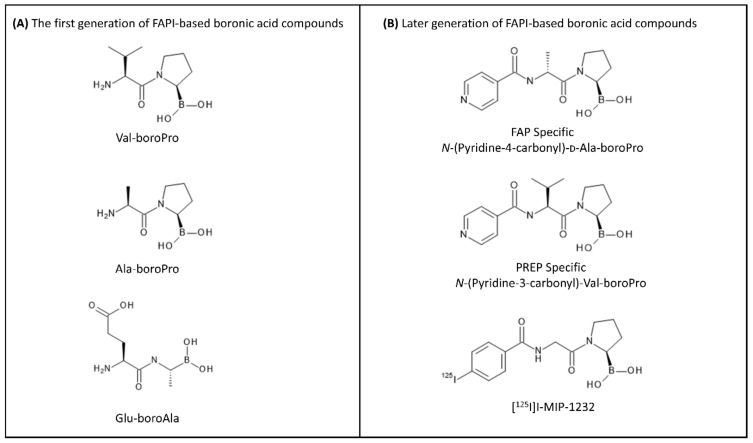
Different relevant FAPI molecules based on boronic acid warhead. (**A**) The first generation of boronic acid-based FAPIs: Val-boroPro, Ala-boroPro, and Glu-boroAla. (**B**) The later generation of FAP-targeted inhibitors: *N*-(Pyridine-4-carbonyl)-D-Ala-boroPro, *N*-(Pyridine-3-carbonyl)-Val-boroPro, and [^125^I]I-MIP-1232.

The first generation of FAP inhibitors was developed based on dipeptide boronic acid inhibitors (Figure 5). Val-boroPro and D-Ala-boroPro have been proved to be highly potent against DPPIV and can be combined with the selectivity to DPP8 and DDP9 in vivo, providing a relatively high therapeutic index above 500 in mouse models [106]. However, the biological behavior of these inhibitors has been hampered due to the lack of their selectivity towards FAP over dipeptidyl peptidases (DPPs) and prolyl oligopeptidase (PREP). In the subsequent work, D-Ala-boroPro was further modified at the amine residue, forming *N*-(Pyridine-4-carbonyl)-D-Ala-boroPro (ARI-3099). ARI-3099 demonstrated low nanomolar potency (IC_50_~36 nM) and high selectivity for FAP (350-fold over PREP), with negligible potency for DPPs in murine FAP (mFAP) transfected human embryonic kidney (HEK) 293 cells [100]. Furthermore, the boronic acid-based FAP inhibitor (MIP-1232) was synthesized and labeled with ^125^I [107]. [^125^I]I-MIP-1232 showed high accumulation in FAP-positive SK-Mel-187 melanoma cells and trivial in an NCI-H69 cell line with low FAP expression. However, the chemical stability and reactivity of boronic acid-based FAP inhibitors towards FAP enzymes are still ambiguous and not well characterized [108]. 

**Figure 6:**
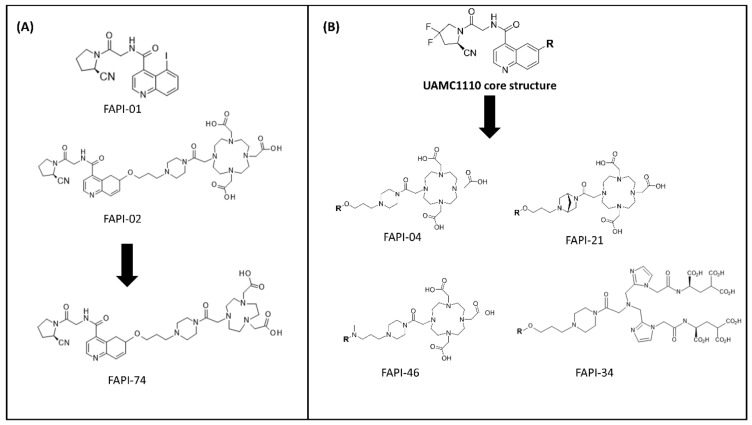
Examples of FAPI molecules developed by the Heidelberg group. (**A**) The first generation of FAPIs (FAPI-01 and FAPI-02) and subsequent work on FAPI-74 using the same FAP-targeting moiety conjugated NOTA chelator. (**B**) Relevant FAPI molecules based on UAMC1110 scaffold, including FAPI-04, FAPI-21, FAPI-34, and FAPI-46.

In 2014, a library of highly potent and selective FAPIs synthesized by utilizing the core of *N*-4-quinolinoyl-Gly-(2*S*)-cyanoPro scaffold as the starting point were vigorously explored in terms of the structure–activity relationship against dipeptidyl peptidases and prolyl endopeptidase [109,110]. The first attempt of radiolabeled quinoline-based FAPIs (i.e., FAPI-01 and FAPI-02) was reported by the Heidelberg research group in Germany [111]. Both FAPIs were synthesized using methods reported earlier by Jansen et al. [109,112]. The basic structure of both compounds consists of a quinoline unit for target selectivity and retention as well as the Gly-Pro motif containing a nitrile group (CN) for a covalent bond formation towards the binding pocket of FAP. FAPI-02 was further designed by the conjugation of the chelator DOTA through a piperazine linker, at position 6 on the aromatic ring of the quinoline group of FAPI, intended to incorporate suitable diagnostic/therapeutic radiometals and to improve the pharmacokinetics (Figure 6A). FAPI-01 and FAPI-02 were radiolabeled with ^125^I and ^68^Ga/^177^Lu, respectively. Although [^125^I]I-FAPI-01 exhibited rapid internalization in both murine FAP-transfected human embryonic kidney (HEK) cells and human FAP-transfected fibrosarcoma HT-1080 cells in vitro, time-dependent efflux and enzymatic deiodination of [^125^I]I-FAPI-01 eliminated its further preclinical evaluation. In contrast, [^68^Ga]Ga-FAPI-02 revealed enhanced binding and uptake in human FAP-expressing cells in vitro and in vivo compared to [^125^I]I-FAPI-01, arising from the enhanced stability of the radiolabeled compound. [^177^Lu]Lu-FAPI-02 biodistribution studies showed the highest uptake at 2 h after administration in human FAP-transfected HT-1080 tumor-bearing mice; however, the retention time of [^177^Lu]Lu-FAPI-02 in the tumor was relatively short and might not be enough to achieve a therapeutic response. Therefore, further efforts of the research group were directed towards the structural modification of FAPI-02 in order to prolong the tumor retention time and improve lipophilicity. The quinoline-Gly-Pro FAPI with a carbonitrile warhead, so-called UAMC1110, reported by Jansen et al. [109] was further functionalized mainly with the chelator DOTA via a variety of linkers (Figure 6B). UAMC1110 exhibits high affinity to FAP compared to PREP (inhibitory potencies (IC_50_); 3.2 nM and 1.8 µM, respectively). On the basic structure of UAMC1110, there is an additional difluoro substitution on the pyrrolidine ring in the Gly-Pro motif, which has been shown to improve target binding affinity [109]. In the first developed series, 13 FAPI derivatives (FAPI-03 to FAPI-15) were synthesized [113]. Briefly, the molecular structures of the FAPI derivatives were designed by attaching various linkers to the quinoline moiety of the UAMC1110 in different positions as well as varying the substitution group (F or H) on the pyrrolidine ring of the Gly-Pro motif. Among all derivatives, FAPI-04 and FAPI-13 were proved to be the most promising tracers compared to others of the same series, demonstrating high binding affinity towards the human FAP-transfected HT-1080 cells with IC_50_ values of 6.5 nM and 4.5 nM, respectively. The in vivo evaluation of [^177^Lu]Lu-FAPI-04 and [^177^Lu]Lu-FAPI-13 was investigated in FAP-transfected HT-1080 xenografts in comparison with previous data of [^177^Lu]Lu-FAPI-02. The uptake in normal tissues was slightly higher for FAPI-04 than FAPI-02, and even higher for FAPI-13. The tumor accumulation of FAPI-04 (3.0%ID/g) and FAPI-13 (4.8%ID/g) was improved compared to FAPI-02 (1.12%ID/g) at 24 h post-injection. However, the tumor-to-blood ratio was more favorable in FAPI-04 (~28) than FAPI-13 (~22) 24 h post-injection. 

Next, another series containing 15 FAPI derivatives was further developed [110]. In this set of compounds, all FAPI derivatives showed equal or better binding affinity compared to FAPI-04. The in vivo pharmacokinetic studies by small-animal PET imaging on FAP-transfected HT-1080 xenografts of the most promising ^68^Ga-labeled candidates of this set (FAPI-21, FAPI-35, FAPI-36, FAPI-46, and FAPI-55), demonstrated rapid tumor accumulation, low background activity, and predominantly renal elimination. In particular, FAPI-36 tended to have a prolonged systemic circulation, resulting in an unfavorable tumor-to-blood ratio and poor imaging contrast. FAPI-21 and FAPI-55 revealed higher uptake in liver and muscle tissues than FAPI-04 while FAPI-35 demonstrated comparable tumor-to-blood and tumor-to-liver ratios with an only slight improvement in tumor-to-muscle ratio. From this series of FAPI tested ligands, FAPI-46 appeared to be the most promising derivative in the series providing the highest tumor-to-background ratios and good tumor accumulation.

^99m^Tc-labeled FAPI tracers for SPECT imaging were also developed from the same group [114]. These attempts led to the generation of FAPI-19 where the UAMC1110 FAP-targeting moiety was linked to a tricarbonyl chelator suitable for radiolabeling with ^99m^Tc. Starting from FAPI-19, several FAPI variants with chelators providing the necessary donor atoms for sufficient coordination of ^99m^Tc and ^188^Re were synthesized, including FAPI-28, FAPI-29, FAPI-33, FAPI-34, and FAPI-43. All the tested compounds exhibited high affinity towards FAP (IC_50_ ranging from 6.4 to 12.7 nM) and a fast internalization rate in FAP-transfected HT-1080 cells. Their scintigraphy studies presented a variable pharmacokinetic performance on FAP-transfected HT-1080 xenografts. Due to the high lipophilicity of the tricarbonyl-^99m^Tc complex, [^99m^Tc]Tc-FAPI-19 revealed high liver uptake and no significant tumor accumulation. As part of the ongoing efforts of the group to reduce the lipophilicity of [^99m^Tc]Tc-FAPI-19 while improving the pharmacokinetic performance, several hydrophilic groups were introduced between the chelator and the inhibitor. Compared to [^99m^Tc]Tc-FAPI-19, other derivatives of this series demonstrated improved tumor uptake and faster clearance from the rest of the body. Overall, [^99m^Tc]Tc-FAPI-34 revealed significant tumor uptake with the lowest accumulation in the liver, biliary gland, and intestine, providing the best in vivo pharmacokinetics among the rest of the same series.

Another ^99m^Tc-labeled FAP-targeting ligand (FL-L3) was lately reported by Roy et al., which includes the radiometal ^99m^Tc coordinated via the ^99m^Tc(V)-oxo moiety while 8-amino-octanoic acid served as the linker between the metal chelator and the FAP inhibitor [115]. [^99m^Tc]Tc-FL-L3 demonstrated high affinity and specificity for FAP when the FAP-transfected human embryonic kidney HEK 293 cell line was used. The in vivo performance of [^99m^Tc]Tc-FL-L3 on MDA-MB231 breast tumor-bearing mice led to specific delineation of the experimental tumor and low non-target uptake.

FAPI-74 is another FAP-specific ligand developed by the Heidelberg group aiming this time at developing a PET tracer suitable for labeling with the two most commonly used PET nuclides in the clinic (^68^Ga and ^18^F). Based on the same FAP-targeting moiety as FAPI-02, FAPI-74 was obtained by exchanging the chelator DOTA with NOTA (1,4,7-triazacyclononane-1,4,7-triacetic acid) (Figure 6A). The superiority of NOTA over DOTA is that it can be easily labeled with ^68^Ga even at room temperature. Furthermore, NOTA can also be labeled with ^18^F via aluminium fluoride (AlF) chemistry, resulting in a more practical large-scale production [116]. To the best of our knowledge, although preclinical data have not been reported yet, the high image contrast and low radiation burden of FAPI-74 PET/CT in 10 patients with lung cancer and rare cancer entities allow its applicability in multiple clinical applications [116,117].

The benefits of radiolabeling with ^18^F over ^68^Ga, such as the higher molar activity of the final radiolabeled product, the improved physical spatial resolution, and the longer half-life that allows sufficient time for quality control and transportation, when necessary, prompt the development of other ^18^F-labeled FAPI-based ligands. FAPI-04 was modified accordingly to achieve radiolabeling with ^18^F. The DOTA on FAPI-04 molecule was substituted with an alkyne group, then further radiolabeled with 6-deoxy-6-[^18^F]F-fluoroglucosyl azide through copper-catalyzed click reaction, forming [^18^F]F-Glc-FAPI [17]. The in vitro radiolabel stability of [^18^F]F-Glc-FAPI in human serum was maintained above 99% at 55 min of incubation. [^18^F]F-Glc-FAPI showed the uptake in the human FAP-transfected HT-1080 cells but lower affinity to FAP (IC_50_ = 167 nM) compared to [^68^Ga]Ga-FAPI (IC_50_ = 32 nM). Additionally, the plasma protein binding and lipophilicity of [^18^F]F-Glc-FAPI were higher than [^68^Ga]Ga-FAPI. In PET studies, tumor uptake of [^18^F]F-Glc-FAPI in human FAP-transfected HT-1080 tumor-bearing mice was higher (4.6%ID/g) compared to [^68^Ga]Ga-FAPI-04 (2.1%ID/g). However, PET images revealed higher tumor-to-background ratios for [^68^Ga]Ga-FAPI-04 due to the lower plasma protein binding and, consequently, faster blood clearance.

**Figure 7:**
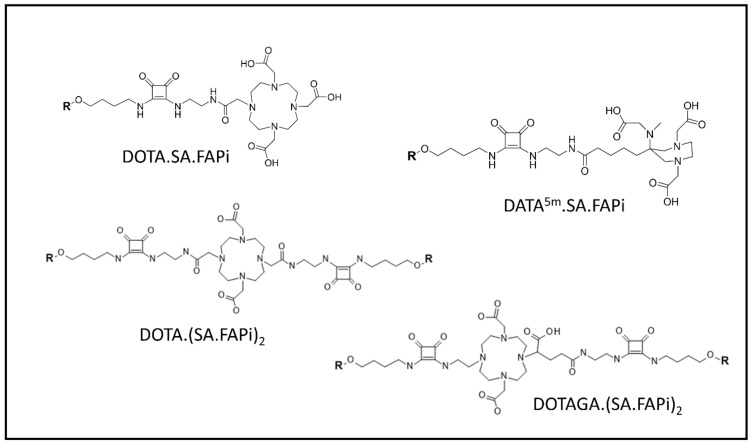
FAPI molecules developed by the Mainz research group: DOTA.SA.FAPi, DATA^5m^.SA.FAPi, DOTA.(SA.FAPi)_2_, and DOTAGA.(SA.FAPi)_2_. ‘R’ represents UAMC1110 scaffold.

On the other hand, the Mainz research group utilized an alternative approach to modify the squaric acid (SA)-based linker bridging between several bifunctional chelators (DATA^5m^, DOTA, and DOTAGA) and the UAMC1110 molecule intended to simplify complex synthetic steps of the current chelator-modified FAPIs (Figure 7) [108,118]. The first generation of precursors, DATA^5m^.SA.FAPi and DOTA.SA.FAPi molecules, were radiolabeled with ^68^Ga and ^177^Lu. Affinity to FAP of the precursors as well as their ^nat^Ga- and ^nat^Lu-labeled derivatives were excellent, resulting in low nanomolar IC_50_ values of 0.7–1.4 nM. Additionally, all the tested compounds showed a low affinity for the related protease PREP (high IC_50_ values of 1.7–8.7 μM). First proof-of-principle in vivo PET-imaging animal studies of one of the tested radioligands, [^68^Ga]Ga-DOTA.SA.FAPi, in HT29 human colorectal xenografts indicated promising results with low background signal and high accumulation in tumor (SUV_mean_ = 0.75), which was higher than [^68^Ga]Ga-FAPI-04 (SUV_mean_ = 0.45) in human FAP-transfected HT-1080 tumors at the same scanning time [108]. However, the prolongation of the residence time of both DATA^5m^.SA.FAPi and DOTA.SA.FAPi in tumor stroma remains a major challenge. Lately, the Mainz group continued to optimize the structure of DOTA.SA.FAPi to enhance tumor uptake and retention time through the formation of dimeric derivatives of DOTA.SA.FAPi. In this second generation, two homodimeric derivatives, DOTA.(SA.FAPi)_2_ and DOTAGA.(SA.FAPi)_2_, were developed through the conjugation of two squaramide ligands with the FAP-targeting moiety [118]. DOTA.(SA.FAPi)_2_ displayed good radiochemical yield (RCY) when radiolabeled with ^68^Ga in the range of 80–90% in 1 M HEPES buffer (pH 5.5) after 10 min of incubation at 95 °C while DOTAGA.(SA.FAPi)_2_ revealed an exceptional RCY (>99%) at the same radiolabeling conditions. Moreover, [^68^Ga]Ga-DOTAGA.(SA.FAPi)_2_ demonstrated good in vitro stabilities in human serum, phosphate-buffered saline (PBS), and isotonic saline solutions where the percentage of intact radiolabeled compound was kept above 90% over 120 min of incubation in the corresponding media. DOTAGA.(SA.FAPi)_2_ was further radiolabeled with ^177^Lu, and the results suggested excellent labeling kinetics in which the RCY was 100% after 5 min of incubation at 95 °C in 1 M ammonium acetate buffer (pH 5.5). The in vitro stability of [^177^Lu]Lu-DOTAGA.(SA.FAPi)_2_ proved to be the best in human serum, resulting in 91% of the radiotracer being intact after 144 h of incubation.

Since most of the FAPI molecules currently referred in the literature contain the chelator DOTA available for radiolabeling with radiometals of oxidation state +3, various SPECT radionuclides, such as ^177^Lu and ^111^In have been also used for radiolabeling and the resulting radiolabeled tracers have been further explored. The first investigation of ^177^Lu-labeled FAPI was carried out with [^177^Lu]Lu-FAPI-02 in comparison to [^125^I]I-FAPI-01 for long-term tracking of pharmacokinetic profiles up to 24 h [111]. The efflux kinetic studies using the FAP-transfected HT-1080 cell line showed that [^177^Lu]Lu-FAPI-02 elimination rate was slower than [^125^I]I-FAPI-01, preserving the intracellular accumulated activity of 12% compared to 1.1%, respectively. The biodistribution studies on human FAP-transfected HT-1080 xenografts showed that the highest uptake of [^177^Lu]Lu-FAPI-02 (4.7%ID/g) was at 2 h, which was gradually decreased to about 1%ID/g at 24 h post-injection. 

Although radiolabeled FAP inhibitors proved to be successful in enabling the imaging of multiple human cancers, the time-dependent clearance from tumors seems to currently limit their utility as FAP-targeted radiotherapeutics. A trifunctional RPS-309 inhibitor synthesized based on the UAMC1110 scaffold was reported in the attempt to improve the therapeutic performance of FAP-based radiotracers [119]. The RPS-309 comprises three active functional moieties: UAMC1110 for FAP targeting, DOTA chelator for radiolabeling, and albumin binding group for the plasma binding that enhances longer retention in plasma. Indeed, [^177^Lu]Lu-RPS-309 demonstrated a prolonged circulation time through the albumin binding group as well as high affinity and retention in liposarcoma SW872 tumor-xenografted mice up to 24 h post-injection. The multifunctional RPS-309 could be a useful tool for the study of the relationship between FAPI structure and substrate activity in the future. In addition, an ultra-high-affinity small organic ligand (OncoFAP), a carboxylic acid moiety, for FAP targeting was very recently reported [120]. The OncoFAP was chemically modified at position 8 on quinoline moiety of the FAPI molecule and further functionalization with the chelator DOTAGA, leading to a FAP-based ligand with a high dissociation constant (*K*_d_) ranging from 0.68 nM for human FAP to 11.6 nM for murine FAP. In biodistribution studies in animal-bearing SK-RC-52.hFAP tumors, [^177^Lu]Lu-DOTAGA-OncoFAP showed the localization into tumors at the maximum dose of 1,000 nmol/kg. Higher than 30%ID/g tumor uptake was achieved within 10 min after administration while the tumor retention was highly sustained at 6 h post-injection (>20%ID/g).

Recently, the fully automated radiosynthesis of ^177^Lu-labeled FAPI-04 and FAPI-46 radiotracer was established. High radiochemical yields and purities of tested ^177^Lu-labeled FAPI products were achieved while the standard and requirements of the European Pharmacopoeia were met [121].

FAPI-04 was further radiolabeled with ^64^Cu (β^+^ emitter) and ^225^Ac (α emitter) for PET imaging and radionuclide therapy, respectively [122]. The biodistribution of ^64^Cu/^68^Ga/^225^Ac-labeled FAPI-04 was conducted in human pancreatic PANC-1 and MIA PaCa-2 xenografts. [^64^Cu]Cu-FAPI-04 showed high accumulation in tumor and most of the normal tissues compared to [^68^Ga]Ga-FAPI-04 in both tumor models. However, high uptake in the liver of [^64^Cu]Cu-FAPI-04 was observed, demonstrating the instability of [^64^Cu]Cu-DOTA complex in vivo. In therapy studies, [^225^Ac]Ac-FAPI-04 was found to significantly suppress tumor growth in the PANC-1 pancreatic cancer xenografts compared to the control group and no severe radiotoxicity was observed; however, the application dose of [^225^Ac]Ac-FAPI-04 in this study was relatively high compared to [^225^Ac]Ac-PSMA-617 therapy (1.5 MBq/kg compared to 50–200 kBq/kg, respectively) in which further investigation on safety, hematologic or renal toxicity should be warranted.

The SPECT radionuclide ^111^In, has also been used for the generation of radiolabeled FAP-based inhibitors for SPECT imaging and potential intraoperative applications. [^111^In]In-QCP02, a novel FAP-targeted SPECT imaging agent was synthesized based on the FAPI-02 analog [123]. The linker between the chelator and the FAP targeting moiety was modified using a more flexible linear hydrocarbon chain containing a semi-rigid piperazine group that allows more efficient internalization at FAP binding sites. [^111^In]In-QCP02 demonstrated a comparable inhibitory effect for FAP with a *K*_d_ value of 16.2 nM and was proved to be more selective to FAP over DPPIV. The serial SPECT/CT imaging studies in animals-bearing U87MG (FAP positive) and PC3 (FAP negative) tumors showed that the uptake of [^111^In]In-QCP02 in U87MG tumor was 4-fold higher than the PC3 tumor at 1 h post-injection. The U87MG tumor could be visualized with [^111^In]In-QCP02 for more than 10 h post-injection while the signal from the radiotracer was already undetectable after 3 h post-injection in the PC3 tumor model. 

### 4.3. Radiolabeled FAP-Targeted Peptides

The targeting of FAP in the tumor stroma using peptide-based radiotracers is still at its outset. By far, there are only two consecutive reports on peptide-targeted radionuclide therapy (PTRT) utilizing FAP as a tumor target [19,20]. FAP-2286 is a FAP-targeted peptidomimetic functionalized with a linker bound to DOTA readily for radiolabeling with ^68^Ga for PET imaging or ^177^Lu for SPECT imaging and radiotherapy. In terms of potency and selectivity, FAP-2286 peptide revealed high affinity within a low nanomolar concentration in both recombinant human FAP protein (~1.1 nM) and cellular FAP-expressing WI 38 fibroblast (~2.7 nM) in vitro [19]. Moreover, FAP-2286 was further labeled with ^177^Lu and its biological behavior was examined in human FAP-transfected HEK 293 and patient-derived sarcoma Sarc4809 xenografts. The tumor uptake of [^177^Lu]Lu-FAP-2286 in FAP-transfected HEK 293 xenografts was about 14%ID/g at 3 h post-injection and the retention remained high over 120 days (~9%ID/g). The antitumor activity revealed that 90% of the animal cohort treated with 60 MBq of [^177^Lu]Lu-FAP-2286 were tumor-free (tumor volume < 10 mm^3^) after 42 days. On the other hand, the biodistribution of [^177^Lu]Lu-FAP-2286 in Sarc4809 xenografts injected at the same radioactivity dose as in the FAP-transfected HEK xenografted mice showed only 5.9%ID/g tumor uptake after 3 h post-injection. After 42 days, a significant tumor suppression was observed compared to the vehicle group; however, the animals were not tumor-free.

Here, an overview of the current status of relevant radiolabeled FAP-targeted tracers is presented (Table 2).

## 5. Clinical Studies of Radiolabeled-Based FAP Inhibitors

As previously described, the clinical study of radiolabeled FAP-based antibodies has been discontinued due to poor pharmacokinetic performance; meanwhile, to the best of our knowledge, so far there is one publication reporting on the clinical assessment of radiolabeled FAP-targeted peptides. The first-in-human report of PTRT of several adenocarcinomas using the radiolabeled peptide [^177^Lu]Lu-FAP-2286 was very recently published [20]. The theranostic feasibility of [^177^Lu]Lu-FAP-2286 was explored in a cohort of 11 patients evaluating the progressiveness and the metastatic rate of pancreatic, breast, ovarian, and colorectal adenocarcinomas. The study indicated a favorable safety use of [^177^Lu]Lu-FAP-2286 with manageable severe side effects in few patients. The post-therapy whole-body scans, including SPECT/CT, showed high tumor accumulation and prolonged retention time of [^177^Lu]Lu-FAP-2286 in all patients at 72 h to 10 days post-injection. Furthermore, the dosimetric studies demonstrated comparable whole-body and bone marrow absorbed dose of [^177^Lu]Lu-FAP-2286 (0.07 and 0.05 Gy/GBq, respectively) to [^177^Lu]Lu-DOTATATE (0.05 and 0.04 Gy/GBq, respectively) and [^177^Lu]Lu-PSMA-617 (0.04 and 0.03 Gy/GBq, respectively). Nevertheless, the limitation of this study remains, including the small and heterogeneous patient cohort, and the dose escalation due to a safety concern from the pre-existing red marrow dysfunction from multiple previous therapies. So far, the reported response rates after PTRT remain improvable, and the combinational radio-immunotherapy might increase the response rate [131] as FAP and CAFs are the main drivers of immune evasion [40,133]. 

Below, we give an overview based on the most relevant clinical trials on FAP-targeting using radiolabeled FAP-based inhibitors, and their performance is discussed mainly in comparison to [^18^F]FDG PET/CT.

The efficacy of [^68^Ga]Ga-FAPI-02, the first generation of FAP inhibitors was investigated in comparison with the reference standard [^18^F]FDG in patients with breast, lung, and pancreatic cancer metastases. The [^68^Ga]Ga-FAPI-02 showed high specific uptake in the primary tumors, lymph nodes, and bone metastases with low background activity while [^18^F]FDG accumulated in most of high glucose consumption organs, such as brain, liver, and spleen. However, the [^68^Ga]Ga-FAPI-02 showed a rapid elimination pattern through the renal clearance and revealed a short tumor retention time, demonstrating about 75% decrease in tumor uptake from 1 to 3 h after tracer administration [111].

**Figure 8:**
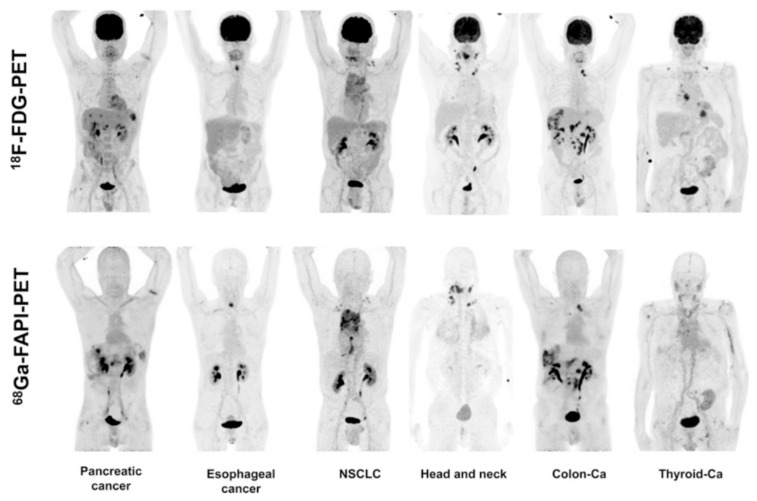
Whole-body PET/CT scans of six selected patients with different tumors entities imaged with [^18^F]FDG and [^68^Ga]Ga-FAPI-02 in the range of a 9-day imaging interval. NSCLC: non-small cell lung cancer; Ca: cancer. The figure is originally published in JNM by Giesel et al. ^68^Ga-FAPI PET/CT: Biodistribution and Preliminary Dosimetry Estimate of 2 DOTA-containing FAP-Targeting Agents in Patients with Various Cancers. J Nucl Med. 2019; 60:386–392. © SNMMI [124].

Continuing the clinical assessment of [^68^Ga]Ga-FAPI-02, PET/CT scans of [^18^F]FDG and [^68^Ga]Ga-FAPI-02 were conducted on six patients with several kinds of cancer (pancreatic, esophageal, lung, head and neck, and colorectal cancer) in a side-by-side comparative study. The observed accumulation of [^18^F]FDG and [^68^Ga]Ga-FAPI-02 in tumors was comparable (average SUV_max_: 7.41 for [^18^F]FDG and 7.37 for [^68^Ga]Ga-FAPI-02; not statistically significant); however, [^68^Ga]Ga-FAPI-02 could not clearly identify primary and metastatic sites in one patient with iodine-negative thyroid cancer. [^68^Ga]Ga-FAPI-02 was found to be superior compared to [^18^F]FDG in terms of background activity, especially in the brain (SUV_max_: 0.32 vs. 11.01), liver (SUV_max_: 1.69 vs. 2.77), and oral/pharyngeal mucosa (SUV_max_: 2.57 vs. 4.88), thus leading to higher contrast of PET images for liver metastases originating from pancreatic and colorectal cancer. It also exhibited a higher ability to delineate esophageal cancer. In the same cohort of patients, it was observed that three out of six patients treated with [^68^Ga]Ga-FAPI-02 could benefit from low tracer accumulation in liver and pharyngeal mucosa, leading to high tumor-to-background ratios (Figure 8).

The improvement in terms of preclinical pharmacokinetic performance of FAPI-04 compared to FAPI-02, was further clinically assessed [124]. The [^68^Ga]Ga-FAPI-04 and [^68^Ga]Ga-FAPI-02 PET/CT scans in two patients with metastasized breast cancer showed that [^68^Ga]Ga-FAPI-04 enhanced tumor retention time by a factor of 3 compared to [^68^Ga]Ga-FAPI-02 at 3 h post-injection. However, both [^68^Ga]Ga-FAPI-04 and [^68^Ga]Ga-FAPI-02 revealed no significant difference in tumor-to-background ratios at 1 h post-injection.

The promising initial clinical data on FAP-directed targeting imaging paved the way for clinical trials with a larger cohort of patients to validate the appropriateness of radiolabeled FAP-based inhibitors as pan-tumor agents. [^68^Ga]Ga-FAPI-04 was further evaluated in 80 patients with 28 different types of tumors (54 primary tumors and 229 metastases) where a robust [^18^F]FDG and other imaging modalities were considered insufficient to obtain the justifiable diagnostic information by physicians [134]. All the enrolled patients were retrospectively identified with histopathologically proven primary tumors or metastases or radiologically definite metastases of histologically proven primary tumors. According to PET/CT scans, the high accumulation of [^68^Ga]Ga-FAPI-04 (SUV_max_ > 12) was presented in cholangiocarcinoma, sarcoma, esophageal, breast, and lung cancers while low uptake (SUV_max_ < 6) was found in renal, thyroid, adenoid cystic, pheochromocytoma, and gastric cancers, indicating that the known [^18^F]FDG limitations in differentiated thyroid and renal cell carcinoma may not be able to be overcome with [^68^Ga]Ga-FAPI. A moderate uptake (SUV_max_ = 6–12) was observed in colorectal, head-and-neck, hepatocellular, pancreatic, ovarian, and prostate cancers. Interestingly, the observed radioactivity in background tissues (e.g., blood pool and muscle) was relatively low, which resulted in improved tumor-to-background ratios. Similarly, another clinical study aimed at comparing the diagnostic efficacy of [^18^F]FDG and [^68^Ga]Ga-FAPI-04 PET/CT in a small cohort of challenging patients with primary and metastatic lesions of several origins [135]. The studied cohort consisted of 75 patients (47 males and 28 females, median age of 61.5 years). All patients were imaged with [^68^Ga]Ga-FAPI-04 while 54 patients with 12 different tumor entities were selected to have a paired scan with [^18^F]FDG in the initial assessment and for the remaining 21 patients, the pairing scan with [^18^F]FDG was performed during the stage of recurrent imaging. [^68^Ga]Ga-FAPI-04 revealed better performance than [^18^F]FDG in terms of detection rate (98.2% vs. 82.1%), especially in hepatocellular, gastric, and pancreatic cancers, which have well-known limitations with regard to the use of [^18^F]FDG PET/CT. These findings indicate that the use of [^68^Ga]Ga-FAPI-04 in PET/CT might be a promising new approach in the case where [^18^F]FDG is limited. Since tumor size has already been found to influence the delineation of primary tumors by [^18^F]FDG due to the partial volume effect and low tumor metabolic activity, [^68^Ga]Ga-FAPI-04 was able to visualize tumors smaller than 1 cm mainly because of high tumor and low background uptake. One more important finding from this study was the significantly higher sensitivity for the detection of lymph node metastases when using [^68^Ga]Ga-FAPI-04 compared to [^18^F]FDG, something which happens due to higher uptake of [^68^Ga]Ga-FAPI-04. Overall, the study demonstrated that [^68^Ga]Ga-FAPI-04 PET/CT has the potential to outperform [^18^F]FDG PET/CT in identifying liver metastases, peritoneal carcinomatosis, brain tumors, and target sites for many types of metastatic cancers. However, the [^68^Ga]Ga-FAPI-04 performance was not more tumor-specific than [^18^F]FDG because [^68^Ga]Ga-FAPI-04 PET was found to yield more false-positive findings.

The two most common types of primary hepatic malignancies, hepatocellular carcinoma (HCC) and intrahepatic cholangiocarcinoma (ICC), are currently identified by MRI and CT or ultrasound imaging techniques and/or biopsy. Although MRI is superior compared to CT/ultrasound in detecting intrahepatic lesions, its use remains limited for the detection of lesions smaller than 2 cm. In addition to this limitation, the molecular characterization of those malignancies may contribute to their therapeutic management. Given the high FAP expression on HCC and ICC in combination with the great promise of [^68^Ga]Ga-FAPI-04 in clinical settings, a pilot clinical study in 17 patients who underwent surgery or biopsy revealed the diagnostic ability of [^68^Ga]Ga-FAPI-04 for detecting and characterizing hepatic nodules in patients with suspected carcinoma. The same study also indicated that patients with less advanced lesions may be more suitable for FAP-targeted imaging compared to those with advanced lesions [136]. Nevertheless, a larger number of patients, as well as a side-by-side comparison with [^18^F]FDG, are essential for the confirmation of these findings. 

Continuing the clinical assessment of the radiolabeled FAP-based inhibitors, FAPI-21 and FAPI-46—the other two outstanding FAPI derivatives that developed in an attempt to improve FAPI-04 performance—indeed, showed superior tumor accumulation and tumor-to-background ratios over FAPI-04 [110]. Although the preclinical data suggested that FAPI-21 provided more specific tumor uptake than FAPI-46, FAPI-21 exhibited high uptake in normal organs in clinical studies of 8 patients with colorectal, ovarian, oropharyngeal, and pancreatic cancers, resulting in lower tumor-to-background ratios compared to FAPI-46. The radiation dosimetry of [^68^Ga]Ga-FAPI-46 was initially evaluated in six cancer patients with cholangiocarcinoma, pancreatic, breast, oropharynx, head-and-neck, and gastric cancers [137]. The mean absorbed radiation dose was determined at three time points after the injection of the radiotracer (10 min, 1 h, and 3 h). After three serial scans, the average effective whole-body absorbed dose of [^68^Ga]Ga-FAPI-46 was about 1.56 mSv with a 200 MBq injected dose, which was lower than other robust ^68^Ga-labeled radiotracers used in the current clinic (e.g., [^68^Ga]Ga-DOTATATE and [^68^Ga]Ga-PSMA-11). The biodistribution study further revealed high tumor-to-background ratios, which were increasing over time, these data suggest superior diagnostic performance and favorable pharmacokinetics. Additionally, a clinical trial comparing the diagnostic performance of [^68^Ga]Ga-FAPI-46 and [^68^Ga]Ga-FAPI-04, and further evaluation of their clinical roles, was performed in 22 patients with lower gastrointestinal tract (LGT) tumors [138]. The radiotracer uptake was quantified by SUV_max_ and SUV_mean_ values. The studies showed that both tracers were able to detect and restage both primary tumors and metastases arising from the LGT, suggesting that these properties may open new possibilities in guiding radiation therapy of LGT tumors. Given the fact that no fasting in patients before the examination is required in combination with the short retention time of the tracers in malignant lesions, FAP targeted imaging agents might be an alternative option for the successful management of patients with LGT and their personalized therapeutic treatment approach. 

FAP imaging using [^68^Ga]Ga-FAPI PET/CT was further investigated in multiple sarcomas [139], gynecological tumors [140], and pancreatic ductal adenocarcinomas (PDAC) [141]. These preliminary clinical data also suggest that FAPI ligands may have the potential to highly contribute to the diagnosis and potential therapy of those types of cancer; however, larger comprehensive studies are required for confirmation.

As ^18^F for various reasons logistically easier to handle, a study was conducted to investigate the biodistribution, radiation dosimetry, and tumor delineation of [^18^F]F-FAPI-74 and [^68^Ga]Ga-FAPI-74 in patients with lung cancer [116]. A high image contrast (SUV_max_ > 10) was observed in tumors, lymph nodes, and metastases after 1 h post-injection. The dosimetric studies in patients showed the normalized effective dose of 1.4 mSv/100 MBq for [^18^F]F-FAPI-74 and 1.6 mSv/100 MBq for [^68^Ga]Ga-FAPI-74, which were lower than [^18^F]FDG (2 mSv/100 MBq). Although [^18^F]FDG PET/CT is considered as the standard radiotracer for staging and target volume delineation in lung cancers, the preliminary experience with 10 patients was not yet sufficient to determine the sensitivity, specificity, and accuracy of [^18^F]F-FAPI-74 PET/CT. Like [^18^F]FDG, [^18^F]F-FAPI-74 PET/CT was able to identify additional distant metastases compared with a diagnostic CT scan. In a case report from the same group, it was shown that [^68^Ga]Ga-FAPI-04 PET/CT delineated brain metastases originated from primary lung cancer [142]. Based on the so far generated data, the FAPI-74 radioligand appears to be a versatile PET radiotracer with the potential for multiple clinical applications.

Besides, ^99m^Tc was applied for FAPI-34 radiolabeling and [^99m^Tc]Tc-FAPI-34 was used for diagnostic scintigraphy and SPECT imaging for the follow-up of [^90^Y]Y-FAPI-46 radiotherapy in patients with ovarian and pancreatic cancers [114]. [^99m^Tc]Tc-FAPI-34 demonstrated high contrast in SPECT/CT images obtained by rapid tumor uptake and fast clearance from the body. The authors also suggested that FAPI-34 could be labeled with ^188^Re (high-energy β^-^ emitter) for radionuclide therapy in future studies. By far, studies on the therapeutic applications of FAPI radiotracers are limited due to the fast washout from the tumors. Although the effort to overcome this obstacle is ongoing, this seems to be one of the biggest challenges on the development of radiotherapeutic FAP-based inhibitors. The first FAP targeted radiotherapeutic application was reported in 2018 [113] when FAPI-04 was labeled with β^-^ emitter ^90^Y. [^90^Y]Y-FAPI-04 exhibited high accumulation in the metastatic site in patients with advanced breast cancer; however, the target occupancy of [^90^Y]Y-FAPI-04 was relatively short (<3 h), eliminating its applicability to achieve the therapeutic response within this time frame. 

[^68^Ga]Ga-DATA^5m^.SA.FAPi, [^68^Ga]Ga-DOTA.SA.FAPi, and [^68^Ga]Ga-DOTAGA.(SA.FAPi)_2_ developed by the Mainz research team were recently investigated for the first time in clinical PET/CT studies. [^68^Ga]Ga-DATA^5m^.SA.FAPi was used to test the restaging of the potential tumor manifestation in a patient with thyroid carcinoma and pulmonary metastasis after treatment with surgery and eight cycles of ^131^I radiotherapy [125]. The decision for the FAP imaging was made because the tumor marker (Tg) was rising and ^131^I whole-body scans were negative. [^68^Ga]Ga-DATA^5m^.SA.FAPi was used instead of [^68^Ga]Ga-DOTA.SA.FAPi for this study due to its slightly better IC_50_ value and a facile preparation based on an instant kit-type protocol. PET/CT scans did not show increased uptake in the neck area and pulmonary lesions but in focal nodular hyperplasia (a benign liver lesion). The observed enhanced tracer uptake demonstrated that [^68^Ga]Ga-DATA^5m^.SA.FAPi PET/CT may be a useful tool to characterize a variety of not only malignant but also benign tumors. Based on this finding, it is important to verify if other benign liver tumors, such as adenomas or hemangiomas, could be detected by this tracer. Moreover, the correlation between [^68^Ga]Ga-DATA^5m^.SA.FAPi PET/CT and Ki-67 as the marker of tumor proliferation, grading, and tumor aggressiveness was investigated in 13 patients with liver metastases of neuroendocrine tumors (NET) [128]. Patients with histologically proven NET from liver biopsy or primary tumor underwent [^68^Ga]Ga-DATA^5m^.SA.FAPi, [^18^F]FDG, and [^68^Ga]Ga-DOTATOC PET/CT scans. In general, 12/13 patients were categorized as positive uptake of [^68^Ga]Ga-DATA^5m^.SA.FAPi and [^68^Ga]Ga-DOTA-TOC in liver metastases while [^18^F]FDG showed the positive uptake only in eight patients. In terms of SUV_max_ and Ki-67 correlation, FDG_SUVmax_ revealed positive correlation coefficient with Ki-67 (rho = 0.543, *p* < 0.05) while DOTATOC_SUVmax_ was in a negative range (rho = −0.618, *p* < 0.05). There was no significant correlation between FAPi_SUVmax_ and Ki-67 (rho = 0.382, *p* > 0.05). However, [^68^Ga]Ga-DATA^5m^.SA.FAPi-positive tumor fraction (FAPi_TF_) demonstrated a strong positive correlation with Ki-67 (rho = 0.770, *p* < 0.01) followed by FDG_TF_ (rho = 0.524, *p* < 0.05) while DOTATOC_TF_ showed a strong negative correlation (rho = −0.828, *p* < 0.01). Besides, the correlation of PET-positive tumor volume with Ki-67 was also determined. [^68^Ga]Ga-DATA^5m^.SA.FAPi-positive tumor volume (FAPi_VOL_) exhibited a moderate correlation coefficient (rho = 0.510, *p* < 0.05), but no significant correlation was observed with FDG_VOL_ and DOTATOC_VOL_ (*p* > 0.05). For dedifferentiation, 2/13 patients had a Ki-67 of above 55% (FAPi_VOL_:DOTATOC_VOL_ ratio > 10) while 11/13 patients had a Ki-67 of max. 40% (max. FAPi_VOL_:DOTATOC_VOL_ ratio = 6.4). Although the results showed that FAPi_VOL_:DOTATOC_VOL_ ratio might enable the prediction of Ki-67 with a high correlation coefficient in patients with NET metastasis in the liver, further study is required with a higher patient population for the assessment of suspicious dedifferentiation and prognosis.

Furthermore, the biodistribution, pharmacokinetics, and dosimetry of [^68^Ga]Ga-DOTA.SA.FAPi, were determined in 54 patients with various cancers while a head-to-head comparison with [^18^F]FDG PET/CT scans was also performed [126]. [^68^Ga]Ga-DOTA.SA.FAPi revealed high target-to-background ratios in various types of cancers while the diagnostic accuracy of [^68^Ga]Ga-DOTA.SA.FAPi was comparable to [^18^F]FDG PET/CT. The results from this study with respect to the selectivity of [^68^Ga]Ga-DOTA.SA.FAPi in various cancers may also contribute to the improvement of the already existing data on FAP molecular imaging. Next, [^68^Ga]Ga-DOTA.SA.FAPi PET/CT-guided [^177^Lu]Lu-DOTA.SA.FAPi radiotherapy was lately reported by the same group [127]. The clinical trial of [^68^Ga]Ga-DOTA.SA.FAPi was conducted in an end-stage breast cancer patient, who had atypical cellular cords and tubules in the fibrotic stroma as shown in the histopathologic examination. [^68^Ga]Ga-DOTA.SA.FAPi provided similar intense matching lesion uptake as observed in [^18^F]FDG PET/CT scan, after which the radionuclide was switched from ^68^Ga to ^177^Lu for radiotherapy. [^177^Lu]Lu-DOTA.SA.FAPi showed a similar biodistribution pattern as [^68^Ga]Ga-DOTA.SA.FAPi where the intense tracer accumulation was found in all lesions. However, this cohort of the study was conducted in only one patient while a larger cohort is required to confirm the uptake pattern of the tracer. The latest clinical report on the new class of FAPI-radiopharmaceuticals utilizing the squaric acid (SA) motif as the linker between the FAP inhibitor and the bifunctional chelator concerning the homodimeric FAP-based inhibitor, DOTAGA.(SA.FAPI)_2_ was also reported [118]. Six patients were recruited for this study who underwent [^18^F]FDG, [^68^Ga]Ga-DOTA.SA.FAPi and [^68^Ga]Ga-DOTAGA.(SA.FAPi)_2_ PET/CT scans, respectively [118]. [^68^Ga]Ga-DOTAGA.(SA.FAPi)_2_ showed an increase in tumor uptake at 1 h post-injection compared to [^68^Ga]Ga-DOTA.SA.FAPi, which was further increased after 3 h post-injection. Thus, the use of dimeric structure DOTAGA.(SA.FAPi)_2_ could be a useful tool to enhance tumor accumulation and retention and may represent an advanced step towards radionuclide FAP targeted therapy. However, [^68^Ga]Ga-DOTAGA.(SA.FAPi)_2_ is accompanied by high, delayed, and heterogeneous blood pool uptake across the patients, thus attributing to a risk of increased radiation dose to the non-target organs. All these findings warrant further investigations in a larger patient population. 

Although the clinical studies are currently in the early stage and prospective studies are ongoing, it is evident that FAP is a highly promising target for cancer imaging and therapy. All the so far acquired PET images utilizing several FAP-based radiolabeled inhibitors document a high tumor-to-background ratio in different tumor subtypes, rendering FAP-based radiotracers as potential pan-tumor imaging agents. FAPI-directed PET/CT seems to play a crucial role in some cancers for which the robust [^18^F]FDG directed PET/CT is known to have limited applications. The FAPI PET/CT provides complementary diagnostic, phenotypic, and biomarker information compared to [^18^F]FDG PET/CT. Since FAP-based inhibitors can also be labeled with therapeutic radionuclides while this is not the case for FDG, this allows research groups to shift their attention to the direction of the theranostic approach where the diagnostic radiotracers will serve as predictive biomarkers for therapeutic responses to FAP-targeted treatments.

## 6. Conclusions

In summary, the critical role of the TME has been recently considered as an integral part of the initiation and progression of tumorigenesis. Monitoring the biological changes in the tumor microenvironment would provide essential information to identify related oncological targets for cancer prevention and treatment. CAFs are of particular interest due to the variety in subtype and phenotype of expressing biomarkers. Among all markers, FAP has been considered as an attractive target that involves in the mediation of immunosuppression in the TME. The introduction of radiolabeled FAP-based inhibitors has led to a new class of radiopharmaceuticals used for visualizing FAP presented in the tumor stroma. Over the past few years, several research groups have demonstrated the potential of FAPI radiotracers in cancer diagnosis and therapy through extensive studies at both preclinical and clinical levels. Based on current reports, FAP imaging with ^68^Ga-labeled FAPI PET/CT appears to be a promising strategy for the visualization of several cancers. However, limitations remain due to the suboptimal specificity of those FAPI molecules to cancer-associated FAP substrate, rapid clearance from the systemic circulation, and short retention time in the tumor. These constraints can hamper the long-term tracking of radiotracers and obstruct the therapeutic efficacy in targeted radionuclide therapy. Therefore, the current effort has been directed towards the structural optimization of FAPI molecules to improve the overall pharmacokinetic properties in vivo. Moreover, the effectiveness of FAP-targeted radiotherapy is ambiguous due to a small number of validated studies while the consolidation with other therapeutic approaches (e.g., immunotherapy and combinatorial molecular radiotherapy) may provide better treatment outcomes.

## Figures and Tables

**Table 1:** Typical biomarker expression in CAFs and their biological identities.

Biomarker	Function	Surface Expression	Related Cancer Model	Shared Expression with Other Cells
Cytoskeleton Marker				
α-SMA	Structure Contractility Motility	No	Pancreatic Liver Breast	Normal fibroblasts Smooth muscle cells Pericytes
S100A4 or FSP-1	Collagen induction Fibrosis Motility	No	Breast	Normal fibroblasts Macrophages Epithelial cells
Vimentin	Structure Motility	No	Breast Prostate	Neurons Epithelial cells Endothelial cells
Membrane-bound protein and receptor				
FAP	Fibrogenesis ECM remodeling	Yes	>90% of all cancers	Stromal fibroblasts Immune cells
PDGFRα/β	Tyrosine kinase activity receptor	Yes	Cervical Colorectal	Normal fibroblasts Skeletal muscle Pericytes Vascular Smooth muscle
ECM Component				
Tenascin C	Cell adhesion	No	Breast Malignant glioma	Cancer cells

**Table 2:** Overview of important radiopharmaceutical-based FAP tracers for nuclear imaging and radiotherapy reported in the literature.

Radionuclide	Inhibitor	Quality of Radiation		Evaluation Phase	Reference
		Imaging	Radiotherapy		
^18^F	FAPI-74	PET	-	Clinical: patients with lung cancer	[116]
	Glc-FAPI-04			Preclinical: fibrosarcoma and glioblastoma xenografts	[17]
^68^Ga	FAPI-02	PET	-	Clinical: various cancers	[124]
	FAPI-04			Clinical: various cancers	[110,111,113,124]
	FAPI-20			Preclinical: fibrosarcoma xenograft	
	FAPI-21			Clinical: various cancers	
	FAPI-22			Preclinical: fibrosarcoma xenograft	
	FAPI-31				
	FAPI-35				
	FAPI-36				
	FAPI-37				
	FAPI-46			Clinical: various cancers	
	FAPI-74			Clinical: patients with lung cancer	
	DOTA.SA.FAPi			Preclinical: colorectal adenocarcinoma xenograftClinical: various cancer patients	[108,125,126,127,128]
	DATA^5m^.SA.FAPi			Preclinical: in vitro modelsClinical: restaging of tumor manifestation, liver tumor and metastases imaging	
	DOTA.(SA.FAPi)_2_			Clinical: patient with thyroid and pancreatic neuroendocrine tumors	[118]
	DOTAGA.(SA.FAPi)_2_				
	RPS-309			Preclinical: liposarcoma xenograft	[119]
^111^In	QCP02	SPECT	-	Preclinical: glioblastoma xenograft	[123]
^99m^Tc	FAPI-34	SPECT	-	Clinical: patients with ovarian metastasis and pancreatic cancer	[114]
	FL-L3			Preclinical: breast cancer xenograft	[115]
^225^Ac	FAPI-04	-	Yes	Preclinical: pancreatic cancer xenograft	[122]
^64^Cu	FAPI-04	PET	Yes		
^177^Lu	FAPI-02	SPECT	Yes	Preclinical: glioblastoma xenograft	[111,129]
	FAPI-04				
	FAPI-46			Fully automated radiosynthesis unit	[121]
	RPS-309			Preclinical: liposarcoma xenograft	[119]
	OncoFAP			Preclinical: renal carcinoma and fibrosarcoma xenografts	[120]
	FAP-2286			Preclinical: HEK-FAP tumor bearing animalsClinical: Patients with diverse adenocarcinomas	[19,20]
^153^Sm	FAPI-46	Scintigraphy	Yes	Clinical: Patient with lung metastatic, fibrous spindle cell soft tissue sarcoma	[130]
^90^Y	FAPI-04	-	Yes	Clinical: metastatic breast cancer patient	[113]
	FAPI-46			Clinical: patient with metastasized breast and colorectal cancers	[131]
				Clinical: patients with metastatic soft tissue or bone sarcoma, and pancreatic cancer	[132]

## Data Availability

Data sharing not applicable.
